# Residual-Based Algorithm for Growth Mixture Modeling: A Monte Carlo Simulation Study

**DOI:** 10.3389/fpsyg.2021.618647

**Published:** 2021-02-26

**Authors:** Katerina M. Marcoulides, Laura Trinchera

**Affiliations:** ^1^Department of Psychology, University of Minnesota, Minneapolis, MN, United States; ^2^NEOMA Business School, Mont-Saint-Aignan, France

**Keywords:** simulation study, growth mixture modeling, latent growth curve models, individual case residuals, unobserved heterogeneity

## Abstract

Growth mixture models are regularly applied in the behavioral and social sciences to identify unknown heterogeneous subpopulations that follow distinct developmental trajectories. Marcoulides and Trinchera (2019) recently proposed a mixture modeling approach that examines the presence of multiple latent classes by algorithmically grouping or clustering individuals who follow the same estimated growth trajectory based on an evaluation of individual case residuals. The purpose of this article was to conduct a simulation study that examines the performance of this new approach for determining the number of classes in growth mixture models. The performance of the approach to correctly identify the number of classes is examined under a variety of longitudinal data design conditions. The findings demonstrated that the new approach was a very dependable indicator of classes across all the design conditions considered.

## Introduction

Growth mixture models are frequently used in the behavioral and social sciences to identify unknown heterogeneous subpopulations in longitudinal data that may each have their own individual growth trajectory. These trajectories can be specified to represent both linear and nonlinear patterns of growth in order to better capture group differences in development and change over time that may be present in the studied processes (Muthén and Shedden, 1999: Grimm and Marcoulides, 2016). For example, an application of growth mixture modeling to longitudinal data on freshman college drinking behaviors detected five different drinking trajectories that markedly fluctuated in their average number of drinks per week and their change over the course of the semester (Greenbaum et al., 2005). By identifying the different drinking growth trajectories, the researchers were subsequently able to classify individuals that were more likely to develop future drinking habits that were problematic.

Recently, Marcoulides and Trinchera (2019) introduced a new modeling method designed to identify groups or classes of individuals that follow similar longitudinal growth trajectories. The method assumes the existence of a fixed but unknown number of latent classes, each of which has a different growth trajectory that is algorithmically determined from the data by clustering individuals with similar patterns of growth based on the individual case residuals (ICRs) (Bollen and Arminger, 1991; Raykov and Penev, 2001). The identified classes essentially represent latent longitudinal segments or strata in which variability is characterized by differences across individuals in the level (intercept) and shape (slope) of their growth trajectories and their corresponding ICRs (Raykov and Penev, 2001, 2002; Haviland et al., 2008; Marcoulides and Trinchera, 2019). Aligned with Wood et al.’s (2015) recommendations that, while there is no truly correct model, we can determine which models are informative, the newly proposed mixture modeling approach was demonstrated as a viable alternative for detecting both the number of classes and the shape of their individual growth trajectories (Marcoulides and Trinchera, 2019).

Traditional growth modeling approaches have been criticized because the determination of the best number of classes must be inferred by the researcher on the basis of their subjective assessment of various model fit criteria (Nagin, 2005). Specifically, traditional growth mixture modeling approaches begin with a fundamental assumption that observed growth trajectories may be comprised of two or more subpopulations, and then use criteria such as the Akaike Information Criterion (AIC) index, the Bayesian Information Criterion (BIC) index, the Vuong-Lo-Mendell-Rubin test, or the bootstrapped parametric likelihood ratio test, and apply relative fit comparisons between fitted models with *k* versus *k* + 1 groupings in order to select the best number of latent classes (Muthén and Muthén, 2014). Unlike traditional growth mixture modeling approaches, in the new modeling approach introduced by Marcoulides and Trinchera (2019) the number of classes are algorithmically determined directly from the data on the basis ICRs (see additional details below).

Although Marcoulides and Trinchera (2019) demonstrated the valuable capabilities of their algorithmic approach and of automating growth mixture model fitting processes with empirical longitudinal data, to date the performance of their approach has not been systematically evaluated. Thus, it remains unclear how well the new approach performs under different growth mixture modeling design conditions, and this ambiguity may ultimately hinder its future use in finite mixture modeling analyses. As model selection in growth mixture modeling is a major concern and has become a highly debated challenge for its effective implementation (Kim, 2014; Liu and Hancock, 2014; He and Fan, 2019), additional work on this topic is clearly needed. To fill this gap, the current research systematically evaluates the performance of the newly proposed Marcoulides and Trinchera (2019) approach to accurately select the correct model (also known as class enumeration) in growth mixture modeling situations. Using Monte Carlo simulation techniques, the current study examines to what extent the newly proposed approach can successfully select the correct latent class model under a variety of longitudinal data design conditions with varying class characteristics and sample sizes. We focus on a number of different growth trajectories with specific model structures and restrict our generalizations of results to the specific conditions we considered.

## Model Notation and Specification

A common growth model assumes change in a set of consecutive observations on a variable *y*, with either equally or unequally spaced assessments at times *t* (Raykov and Penev, 2014)^1^. If we assume a mean vector μ and the covariance matrix **Σ** of the observed variables *y* are a function of the **Λ** factor loadings, the η latent variable means, the latent **Ψ** variance-covariance matrix, and a **Θ** residual variance-covariance matrix (e.g., Raykov and Marcoulides, 2006), the model can be specified as:

(1) $$\Sigma =\Lambda \,\Psi \,\Lambda^{\prime }+\Theta$$

(2) μ=Λ⁢η

Then assuming uncorrelated residuals and homoscedasticity, we can define

(3) $$\Lambda =\left[\begin{array}{cc} 1 & t_{1}\\ 1 & t_{2}\\ 1 & \vdots \\ 1 & t_{T} \end{array}\right]$$

(4) $$\Psi =\left[\begin{array}{cc} \sigma_{\eta_{0}}^{2} & \sigma_{\eta_{0}\,\eta_{1}}\\ \sigma_{\eta_{0}\,\eta_{1}} & \sigma_{\eta_{1}}^{2} \end{array}\right]$$

(5) $$\eta =\left[\begin{array}{c} \eta_{0}\\ \eta_{1} \end{array}\right]$$

(6) $$\Theta =\left[\begin{array}{ccc} \sigma_{e}^{2} & 0 & 0\\ 0 & \ddots & 0\\ 0 & 0 & \sigma_{e}^{2} \end{array}\right]$$

where the number of measurement occasions *T* at times *t*_1_ to *t*_*T*_, residual variance $\sigma_{e}^{2}$, mean η_0_ and variance $\sigma_{\eta_{0}}^{2}$ of the latent intercept, mean η_1_ and variance of the latent slope $\sigma_{\eta_{1}}^{2}$, and covariance of the latent intercept-slope σ_η_0_η_1__. One approach that is frequently used to estimate this growth model is via the latent basis growth model (also commonly referred to as the level and shape model). This approach is frequently used because it often results in more optimal functional forms of the observed data (Raykov and Marcoulides, 2006; Kwok et al., 2010). To apply a latent basis growth model, the value of *t*_1_ is fixed to 0 so that the initial status of ***y*** is the reference point and the last occasion *t*_*T*_ is fixed to 1, while all other values are freely estimated so as to capture the change between the first and last measurement occasions (McArdle, 1988; Raykov and Marcoulides, 2006).

A variety of other choices are also possible for the values of *t*_1_ through *t*_*T*_ all of which may be accomplished by changing the matrix of factor loadings **Λ** (Marcoulides and Khojasteh, 2018). For example, the linear intercept-slope model would fix the value of *t*_1_ to 0, the value of *t*_2_ to 1, *t*_3_ to 2, and so on in increasing order, up to the last measurement occasion *T*. In addition to decisions about the structure of the factor matrix **Λ**, choices about the residual matrix **Θ** can also be made when fitting latent growth models; although the convention of assuming a single fixed residual variance ($\sigma_{e}^{2})$over time and fixed zero covariances between measurement occasions is the most commonly adopted (Marcoulides, 2019).

If in the population there are *K* number latent classes with unknown sizes (where each class is assumed to be mutually exclusive and collectively exhaustive), then the model in Eqs 1–6 can be subsequently specified for *k = 1,…, K* latent classes. The traditional growth model is thereby extended and allows latent classes with potentially differing developmental trajectories to be considered (for additional technical details see, Nagin, 2005; Muthén and Muthén, 2014; Marcoulides and Trinchera, 2019). These models examine how the classes differ and whether there is also individual intercept and slope variability in the growth trajectory within each class.

Marcoulides and Trinchera (2019) proposed an iterative approach for algorithmically detecting unobserved heterogeneity in latent growth models as given in Eqs 1–6 using ICRs that represented the differences between a person’s observed score and their predicted score, which is determined via the Bartlett factor score method (Bollen and Arminger, 1991; Coffman and Millsap, 2006; Raykov and Penev, 2014). The algorithm utilized a closeness measure to determine the distance between observations and the global and each of their corresponding local models. This approach assumes if a latent class exists, then all the observed scores in within the same latent class would follow similar growth trajectories and have similar ICRs based on the global model. Thus, if an observed score was correctly assigned to the appropriate latent class, the ICR with respect to the local model that was determined for that particular latent class would be minimized compared to any of the other possible local models.

Marcoulides and Trinchera (2019) applied a closeness measure (*CM*_*ik*_) for individual *i* in local latent growth model for class *k* that was defined as:

(7) $$C\,M_{i\,k}=\sqrt{\frac{\frac{\sum_{j=1}^{J}\sum_{t=1}^{T}\left[\varepsilon_{i\,t\,j\,k}^{2}/c\,o\,m\,\left(\eta_{j\,k},y_{t\,j}\right)\right]}{\sum_{i=1}^{N}\sum_{j=1}^{J}\sum_{t=1}^{T}\left[\varepsilon_{i\,t\,j\,k}^{2}/c\,o\,m\,\left(\eta_{j\,k},y_{t\,j}\right)\right]}}{N-2}}$$

where *T* is the number of *y* measured variables for individual *i*, *J* is the number of factors in the growth model (e.g., the intercept-slope model estimates the intercept and the slope factors, therefore *J* is equal to 2), *N* is the total number of observations, $\varepsilon_{i\,t\,j\,k}^{2}$ is the ICR for observation *i* for the *t*-th variable in the modeled factors in the local growth model *k*. Finally, *com*(η_*jk*_,*y*_*tj*_) is the communality estimate which can be computed as, for each latent class *k*, the mean of the squared correlation between the measured variable *y* and its corresponding estimated latent variable score.

Based on the size of this closeness measure *CM*_*ik*_ for each individual relative to each local model, individuals were assigned to a particular local growth model based on their smallest *CM*_*ik*_ value. The individuals that comprise the latent classes was then updated and a set of potentially new local growth models was estimated. This process continues iteratively until the stopping criterion, that <5% of the individuals are changing their class membership from one iteration to the next, was met. Once this class enumeration process stabilizes, the final local growth models were estimated, enabling the parameter estimates and growth trajectories for each latent class could be evaluated. This residual-based algorithm for identifying and modeling unobserved heterogeneity in latent growth models was shown to both identify groups of individuals that follow similar growth trajectories and simultaneously determine their class-specific parameters.

## Method

### Monte Carlo Data Simulation

In order to systematically evaluate the performance of the Marcoulides and Trinchera (2019) approach to accurately select the correct model in growth mixture modeling situations, we simulated data using various longitudinal data design conditions. We simulated data using the Monte Carlo simulation techniques available in M*plus* (Muthén and Muthén, 2014). The Marcoulides and Trinchera (2019) approach was implemented using the software program R (R Development Core Team, 2011) using the *lavaan* package (Rosseel, 2012) to fit the growth models^2^.

Based on a detailed review of the literature on past growth mixture modeling simulation studies, we fixed a number of factors across all data design conditions while others were varied (e.g., Lubke and Muthén, 2005, 2007; Nylund et al., 2007; Wang and Bodner, 2007; Peugh and Fan, 2012; He and Fan, 2019). Parameters that were fixed in the simulations included the number of measurement occasions, the degree of class separation, the mixing ratio, the variance and covariance for the intercept and slope, and the residuals for the repeated measurement occasions. These fixed design conditions were set up in accordance with those most commonly adopted in previous simulation studies. Specifically, treating the studies by Nylund et al. (2007) and He and Fan (2019) as a general guide, four repeated measurement occasions were used, the mixing ratio was set at *N*/*k*so that each class had the same number of observations, the variance and covariance among the growth factors were set at $\sigma_{\eta_{0\,i}}^{2}$ 0.25, $\sigma_{\eta_{1\,i}}^{2}$ 0.04, and σ_η0_*i*_η1_*i*__ 0, the residual structure was specified at $\sigma_{1}^{2}$ 0.15, $\sigma_{2}^{2}$ = 0.20, $\sigma_{3}^{2}$ = 0.20, and $\sigma_{4}^{2}$ = 0.35, while the degree of class separation was set at a Mahalanobis Distance (MD) value of 1. Although many growth models are frequently fit following the convention of assuming that there is a single residual variance over time, imposing such a constraint on residual variance is quite arbitrary. The fixed values of the residual variances selected in this study were based on residual structures commonly encountered in empirical longitudinal data (as suggested by Peugh and Fan, 2012 and He and Fan, 2019). In terms of the selected class separation, it is important to note that although past research has suggested that the degree of class separation quantified using MD should generally be larger than 2, to accurately identify latent classes in growth mixture models even when there is a relatively large sample size (e.g., Lubke and Muthén, 2007; Tolvanen, 2007; Tofighi and Enders, 2008; Peugh and Fan, 2012; Liu et al., 2014), in this study we elected to use a fixed class separation level of MD = 1, a value that is not expected to provide well-separated classes. This small separation level was therefore selected in order to examine the performance of the new approach under what were considered by Tofighi and Enders (2008) to be the most severe low class separation conditions.

Parameters that were varied in the simulations included the number of true classes in the population, the sample size, and the shapes of the latent growth trajectories. These design elements were also selected based on previous simulation studies that had identified each element as potentially impacting class enumeration in growth mixture modeling (Nylund et al., 2007; Peugh and Fan, 2012). Specifically, the number of true classes for the population were stipulated to range from two to four classes, the sample sizes were set at *N* = 180, *N* = 540, and *N* = 1080 observations (to reflect small, medium, and large samples), and the shapes of the latent trajectories were specified to reflect different-intercept-same-slope and different-intercept-different-slope growth trajectory conditions.

A total of 100 replications were drawn for each population model and design condition^3^. Although the number of replications is generally selected arbitrarily, we followed the recommendations provided by He and Fan (2019) and Kim (2014) who indicated that 100 replications are adequate to provide sufficient reliability in the summary information calculated in growth mixture modeling studies, especially those expected to have high convergence rates. Similar recommendations with respect to the number of replications were also given by Nylund et al. (2007), particularly when the algorithmic burden that might be required to calculate the residuals and perform the class enumerations is extensive^4^.

To evaluate the accuracy of the Marcoulides and Trinchera (2019) approach, a performance rate measure was defined as the percentage of the total replications where the true number of latent classes was correctly identified. High percentage values would indicate that the approach was able to correctly identify the true class growth model that generated the data. The percentage rate measure can be considered similar to ones that have been used in other simulation studies that were also trying to detect the true number of classes (e.g., Nylund et al., 2007; Tofighi and Enders, 2008). As indicated by Nylund et al. (2007), because classes are habitually used as a key characteristic for interpreting results and making inferences, the accurate determination of the number of classes in mixture modeling is considered the most critical issue in the application of these models. Accordingly, the goal of this simulation study is to investigate the performance of the new approach to correctly identify the number of classes examined using the above described design conditions. We also note that because the new approach is entirely automated, we do not compare its performance to traditional growth mixture modeling approaches. This is because in traditional approaches the determination of the number of classes is based on subjectively applying various fit criteria that regularly provide divergent results (e.g., Nylund et al., 2007; He and Fan, 2019). Thus there is to date no generally accepted fit criteria for determining the correct number of classes in traditional approaches, and oftentimes the best fit criteria may highly depend on the specific design conditions (Grimm et al., 2017). Given that model selection in traditional growth mixture modeling remains a major concern and the most prominent and disputed challenge for its application (He and Fan, 2019), in this simulation study we only investigate the performance of the proposed new approach to correctly identify the number of classes examined without any comparison to other methods. Such a comparison between methods would ultimately be problematic and akin to comparing the performance of an unsupervised data mining technique to a supervised technique – one approach is entirely algorithmically driven and the other involves a user interfacing with the approach.

## Results

Table 1 provides the proportion of replications where the true latent class model was correctly identified in the simulated longitudinal data designs. The corresponding standard errors for the proportion of replications where the latent class model was correctly identified are also provided^5^. An examination of the various percentage rates and their standard errors displayed in Table 1 indicates multiple important findings. First, it is clear that this proposed approach generally performed consistently well across all the examined conditions. Also noteworthy to mention is the fact that although we elected to examine the approach using what is generally considered a severe low class separation condition, class separation did not appear to impact the detection of latent classes. Indeed, given the obtained results under the examined low class separation condition, we would expect the approach to perform even better in situations with well-separated classes.

**TABLE 1:** Percentage rate where the true number of latent classes was correctly identified.

	Sample size *(N)*	180	540	1080
Model 1	**Number of classes (*K*)**			
	2	80 (0.029)	95 (0.009)	98 (0.004)
	3	75 (0.032)	92 (0.010)	96 (0.006)
	4	60 (0.036)	90 (0.012)	95 (0.006)

Model 2	**Number of classes (*K*)**			

	2	85 (0.026)	96 (0.008)	99 (0.003)
	3	81 (0.029)	94 (0.010)	96 (0.006)
	4	72 (0.033)	93 (0.011)	96 (0.006)

*Model 1, different-intercept-same-slope growth trajectories. Model 2, different-intercept-different-slope growth trajectories. Values in parentheses correspond to the standard errors of the percentage rates where the true model was correctly identified.*

Second, the overall performance accuracy of the approach was to some degree influenced by sample size. For example, with small sample sizes (*N* = 180) the average percentage rates were consistently lower (in the range of 60–85%) than when models with medium (*N* = 540) and large (*N* = 1080) sample sizes were analyzed (in the range of 90–99%). This same pattern of results was observed in models where the shapes of the latent trajectories were specified to reflect different-intercept-same-slope and models with different-intercept-different-slope growth trajectories, although those with different-intercept-different-slope growth trajectories appeared to be slightly easier to accurately detect.

Third, the number of classes was also an important factor that influenced the overall performance rate of the approach. Models with a larger number of latent classes were more difficult to accurately detect, although the level of accuracy of the approach increased considerably when the same models were analyzed with medium and large sample sizes. This same pattern of results concerning the influence of the number of classes was again observed in models where the shapes of the latent trajectories were specified to reflect different-intercept-same-slope and models with different-intercept-different-slope growth trajectories. We also note that there was not a single instance in which the algorithm over-extracted the number of classes. Overall, the examined approach showed a consistently high percentage for correctly identifying the number of latent classes when the sample sizes were large (ranging from a low of 90% to a high of 99%). With smaller sample sizes, the percentage for correctly identifying the number of latent classes was much lower, ranging from a low of 60% to a high of 85%.

## Discussion

This article examined the performance of a new algorithmic approach that can be used to identify the presence of unknown latent classes in growth mixture models. The algorithm is particularly useful to researchers interested in studying developmental processes where there may be unobserved differences in development, or change over time in longitudinal data. The approach algorithmically clusters individuals who follow the same estimated growth trajectory by evaluating their ICRs. Although the benefits of using the new approach had been previously illustrated using empirical data by Marcoulides and Trinchera (2019), its performance under a variety of data design conditions had not been systematically evaluated. Thus, it was uncertain how well the new approach would perform under different growth mixture modeling design conditions. As model selection in growth mixture modeling is one of the most prominent and debated challenge for its effective application, additional work on this topic was needed.

Using Monte Carlo simulation techniques, the current study examined to what extent the newly proposed approach could successfully select the correct latent class model under a variety of longitudinal data design conditions with contrasting class characteristics and sample sizes. We generated data under a range of different modeling conditions that included both fixed and variable parameters, all of which were set up in accordance with those most commonly adopted in past simulation studies on class enumeration in growth mixture modeling. Parameters that were fixed in the simulations included the number of measurement occasions, the degree of class separation, the mixing ratio, the variance and covariance for the intercept and slope, and the residuals for the repeated measurement occasions. Parameters that were varied included the number of true classes in the population, the sample size, and the shapes of the latent growth trajectories.

Overall, the proposed approach was found to perform consistently well in detecting the true latent classes across all the examined conditions, even those involving severe low class separation conditions, which past research has shown can be a particularly problematic condition (Peugh and Fan, 2012). Although the overall accuracy of the approach was to a degree influenced by small sample sizes, its performance improved when medium and large sample sizes were analyzed. Further studies are still necessary to bring about a better understanding of the sensitivity of the approach to small sample sizes, but for now it is evident that medium to large sample sizes can be effectively studied. This same pattern of results was observed in models where the shapes of the growth trajectories were specified to include different-intercept-same-slope and different-intercept-different-slope trajectories. Thus, it would appear that in general the shapes of the latent trajectories do not influence the detection of heterogeneous classes. The number of classes was also found to be a factor that impacted the precision of the approach, although overall accuracy again increased with medium and large sample sizes. In summary, the results of this study demonstrated the excellent capabilities of the Marcoulides and Trinchera (2019) approach and showed that it can be effectively used as a reliable and accurate tool for determining the correct number of classes in growth mixture models.

Although we have only touched the surface of the different types of growth mixture modeling designs on which the new approach might be applied in order to address some developmental inquiries, the findings of this study are very promising. As one should be with all simulation studies, we must also be cautious about generalizing these findings to conditions beyond those specifically examined in this study. At the very least, we hope that researchers will contemplate using the approach in future finite mixture modeling analyses. The potential utility of the new approach for further understanding group differences in developmental processes and detecting unobserved heterogeneity is excellent and we encourage researchers to examine further the application of this approach in their own research. An important challenge for the foreseeable future will involve applying this new method to diverse data conditions and models to further establish its effectiveness as a mixture modeling tool.

## Data Availability Statement

The datasets presented in this study can be found in online repositories. The names of the repository/repositories and accession number(s) can be found below: https://sites.google.com/umn.edu/marcoulides-data-analytics/data-code.

## Author Contributions

KM led the writing and computations, and finalized the manuscript. LT contributed to computations and the writing of the manuscript. Both authors contributed to the article and approved the submitted version.

## Conflict of Interest

The authors declare that the research was conducted in the absence of any commercial or financial relationships that could be construed as a potential conflict of interest.

## Footnotes

1 For simplicity in model specification, we assume that there are not any necessary covariates to be included, although we could of course extend the model to include covariates.

2 The complete R source code and the description of the various steps necessary to implement the proposed iterative algorithm are available in Marcoulides and Trinchera (2019).

3 The programming code for the simulated data is available on the first author’s website: https://sites.google.com/umn.edu/marcoulides-data-analytics/data-code.

4 We note that effectively measuring algorithmic efficiency is surprisingly difficult (Kreiberg et al., 2020). For example, one can count the number of iterations and/or evaluate estimation time until a solution is reached. However, the specific hardware processor used and allocated resources assigned at any time the processor is busy with ongoing processes are key components that ultimately lead to variation in the number of iterations and estimation time. Thus, different hardware processors may be expected to provide diverse iteration and timing results. In this study, using the 64-bit operating system on an Intel Core i7-8700 CPU at 4.6 Ghz the iteration and estimation timing results ranged from a low of 92 iterations in 23 s to a high of 671 iterations in 2820 s. The iteration and timing results were longer for more complex models with larger samples.

5 As indicated by Nylund et al. (2007) and Muthén and Muthén (2002), the ability to recover population parameters that generated the simulated data can additionally be evaluated by examining coverage estimates (with the general rule of thumb that good coverage estimates should be between 0.91 and 0.98). While coverage values are not reported in the results presented here, they were all found to be very good.

## References

[B1] Bollen K. A. Arminger G. (1991). Observational residuals in factor analysis and structural equation models. *Sociol. Methodol.* 21 235–262. 10.2307/270937

[B2] Coffman D. L. Millsap R. E. (2006). Evaluating latent growth curve models using individual fit statistics. *Struct. Equat. Model.* 13 1–27. 10.1207/s15328007sem1301_1

[B3] Greenbaum P. Del Boca F. Darkes J. Wang C.-P. Goldman M. (2005). Variation in the drinking trajectories of freshmen college students. *J. Consult. Clin. Psychol.* 73 229–238. 10.1037/0022-006x.73.2.229 15796630

[B4] Grimm K. G. Marcoulides K. M. (2016). Individual change and the timing and onset of important life events: methods, models, and assumptions. *Int. J. Behav. Dev.* 40 87–96. 10.1177/0165025415580806

[B5] Grimm K. G. Mazza G. L. Davoudzadeh P. (2017). Model selection in finite mixture models: a k-fold cross-validation approach. *Struct. Equat. Model.* 24 246–256. 10.1080/10705511.2016.1250638

[B6] Haviland A. Rosenbaum P. R. Nagin D. S. Tremblay R. E. (2008). Combining group-based trajectory modeling and propensity score matching for causal inferences in nonexperimental longitudinal data. *Dev. Psychol.* 44 422–436. 10.1037/0012-1649.44.2.422 18331133

[B7] He J. Fan X. (2019). Evaluating the performance of the k-fold cross-validation approach for model selection in growth mixture modeling. *Struct. Equat. Model.* 26 66–79. 10.1080/10705511.2018.1500140

[B8] Kim S.-Y. (2014). Determining the number of latent classes in single- and multi-phase growth mixture models. *Struct. Equat. Model.* 21 263–279. 10.1080/10705511.2014.882690 24729675 PMC3979564

[B9] Kreiberg D. Marcoulides K. M. Olsoon U. H. (2020). A faster procedure for estimating CFA models applying minimum distance estimators with a fixed weight matrix. *Struct. Equat. Model.* 10.1080/10705511.2020.1835484 [Epub ahead of print].

[B10] Kwok O.-M. Luo W. West S. G. (2010). Using modification indexes to detect turning points in longitudinal data: a monte carlo study. *Struct. Equat. Model.* 17 216–240. 10.1080/10705511003659359

[B11] Liu M. Hancock G. R. (2014). Unrestricted mixture models for class identification in growth mixture modeling. *Educ. Psychol. Measur.* 74 557–584. 10.1177/0013164413519798

[B12] Liu Y. Luo F. Liu H. (2014). Factors of Piecewise growth mixture model: distance and pattern. *Acta Psychol. Sin.* 46 1400–1412. 10.3724/sp.j.1041.2014.01400

[B13] Lubke G. Muthén B. O. (2007). Performance of factor mixture models as a function of model size, covariate effects, and class-specific parameters. *Struc. Equat. Model.* 14 26–47. 10.1080/10705510709336735

[B14] Lubke G. H. Muthén B. O. (2005). Investigating population heterogeneity with factor mixture models. *Psychol. Methods* 10 21–39. 10.1037/1082-989x.10.1.21 15810867

[B15] Marcoulides K. M. (2019). Reliability estimation in longitudinal studies using latent growth curve modeling. *Measurement* 17 67–77. 10.1080/15366367.2018.1522169

[B16] Marcoulides K. M. Khojasteh J. (2018). Analyzing longitudinal data using natural cubic smoothing splines. *Struc. Equat. Model.* 25 965–971. 10.1080/10705511.2018.1449113

[B17] Marcoulides K. M. Trinchera L. (2019). Detecting unobserved heterogeneity in latent growth curve models. *Struc. Equat. Model.* 26 390–401. 10.1080/10705511.2018.1534591

[B18] McArdle J. J. (1988). “Dynamic but structural equation modeling of repeated measures data,” in *Handbook of multivariate experimental psychology*, 2nd Edn, eds Cattell R. B. Nesselroade J. (New York, NY: Plenum).

[B19] Muthén B. O. Shedden K. (1999). Finite mixture modeling with mixture outcomes using the EM algorithm. *Biometrics* 55 463–469. 10.1111/j.0006-341x.1999.00463.x 11318201

[B20] Muthén L. Muthén B. O. (2002). How to use a Monte Carlo study to decide on sample size and determine power. *Struc. Equat. Model.* 9 599–620. 10.1207/s15328007sem0904_8 33486653

[B21] Muthén L. K. Muthén B. O. (2014). *Mplus User’s Guide.* Los Angeles, CA: Muthén & Muthén.

[B22] Nagin D. S. (2005). *Group-Based Modeling of Development.* Cambridge, MA: Harvard University Press.

[B23] Nylund K. L. Asparouhov T. Muthén B. O. (2007). Deciding on the number of classes in latent class analysis and growth mixture modeling: a monte carlo simulation study. *Struc. Equat. Model.* 14 535–569. 10.1080/10705510701575396

[B24] Peugh J. Fan X. (2012). How well does growth mixture modeling identify heterogeneous growth trajectories? A simulation study examining GMM’s performance characteristics. *Struct. Equat. Model.* 19 204–226. 10.1080/10705511.2012.659618

[B25] R Development Core Team (2011). *R: A Language and Environment for Statistical Computing.* Vienna: R Foundation for Statistical Computing.

[B26] Raykov T. Marcoulides G. A. (2006). *A First Course in Structural Equation Modeling*, 2 Edn. Mahwah, NJ: Lawrence Erlbaum Associates Inc.

[B27] Raykov T. Penev S. (2001). “The problem of equivalent structural equation models: an individual residual perspective,” in *New Developments and Techniques in Structural Equation Modeling*, eds Marcoulides G. A. Schumacker R. E. (Mahwah, NJ: Erlbaum), 297–322.

[B28] Raykov T. Penev S. (2002). “Exploring structural equation model misspecifications via latent individual residuals,” in *Latent Variable and Latent Structural Models*, eds Marcoulides G. A. Moustaki I. (Mahwah, NJ: Lawrence Erlbaum), 121–134.

[B29] Raykov T. Penev S. (2014). Latent growth curve model selection: the potential of individual case residuals. *Struc. Equat. Model*. 21 20–30. 10.1080/10705511.2014.856693

[B30] Rosseel Y. (2012). Lavaan: an R package for structural equation modeling. *J. Statist. Softw.* 48 1–36. 10.1002/9781119579038.ch1

[B31] Tofighi D. Enders C. K. (2008). “Identifying the correct number of classes in growth mixture models,” in *Advances in Latent Variable Mixture Models*, eds Hancock G. R. Samuelsen K. M. (Greenwich, CT: Information Age Publishing, Inc.), 317–341.

[B32] Tolvanen A. (2007). *Latent Growth Mixture Modeling: A Simulation Study.* Unpublished doctoral dissertation, University of Jyväskylä, Jyväskylä.

[B33] Wang M. Bodner T. E. (2007). Growth mixture modeling: identifying and predicting unobserved subpopulations with longitudinal data. *Organ. Res. Methods* 10 635–656. 10.1177/1094428106289397

[B34] Wood P. K. Steinley D. Jackson K. M. (2015). Right-sizing statistical models for longitudinal data. *Psychol. Methods* 20 470–488. 10.1037/met0000037 26237507 PMC4679639

